# Comprehensive and Integrative Analysis of Two Novel SARS-CoV-2 Entry Associated Proteases CTSB and CTSL in Healthy Individuals and Cancer Patients

**DOI:** 10.3389/fbioe.2022.780751

**Published:** 2022-01-26

**Authors:** Yongbiao Huang, Shiyu Li, Shanshan Huang, Jingyao Tu, Xinyi Chen, Lingyan Xiao, Bo Liu, Xianglin Yuan

**Affiliations:** Department of Oncology, Tongji Hospital, Tongji Medical College, Huazhong University of Science and Technology, Wuhan, China

**Keywords:** SARS-CoV-2, CTSB, CTSL, cancer, immune infiltration

## Abstract

More than 200 million people have been infected with severe acute respiratory syndrome coronavirus 2 (SARS-CoV-2) and 4 million deaths have been reported worldwide to date. Cathepsin B/cathepsin L (CTSB/L) are SARS-CoV-2 entry–associated proteases and facilitate SARS-CoV-2 to infect host cells. However, the expressions of CTSB/L in healthy individuals and cancer patients remain not fully elucidated yet. Here, we comprehensively profiled the expressions and distributions of CTSB/L in human normal tissues, cancer tissues, and cell lines. Moreover, we compared CTSB/L expressions between various cancers and matched normal tissues, and investigated their genetic alteration and prognostic values in pan-cancer. Finally, we also explored the correlation between CTSB/L expressions and immune infiltration. We found that CTSB was highly expressed in most tissues, and CTSL was highly expressed predominantly in the digestive, urinary, and respiratory systems, such as the lungs, liver and gallbladder, and kidney tissues in the translational level. Moreover, cancer patients may be more susceptible to SARS-CoV-2 infection. Our data suggested that CTSB/L are overexpressed in aerodigestive and genitourinary cancers when compared with that in matched normal tissues, and their expressions were closely related to the prognosis of some cancer types. Interestingly, CTSB/L expressions were significantly correlated with immune cell infiltration in manifold cancer tissues and their corresponding normal tissues. In conclusion, our study shows a comprehensive bioinformatic analysis of two important SARS-CoV-2 entry–related proteases, which could provide a potential indication on prevention of SARS-CoV-2 infection.

## Introduction

Severe acute respiratory syndrome coronavirus 2 (SARS-CoV-2) is considered as a new human-infecting betacoronavirus, which has resulted in an outbreak of coronavirus diseases 2019 (COVID-19) worldwide (Lu et al., 2020; Zhu et al., 2020). As of December 19, 2021, more than 270 million cases and five million deaths have been reported, which has been a horrible threat to global human health (www.worldometers.info/coronavirus/). It is well known that the spike glycoprotein of SARS-CoV-2 can bind the angiotensin-converting enzyme 2 to facilitate its entrance into the host cells (Hoffmann et al., 2020; Lan et al., 2020). Exposed to the external environment, the aerodigestive and genitourinary tracts may provide potential routes for SARS-CoV-2 transmission.

In addition, some host cell proteases such as cathepsin B (CTSB) and cathepsin L (CTSL) can cleave and activate the spike protein, leading to the virus infection (Smieszek et al., 2020; Bollavaram et al., 2021). It has been demonstrated that SARS coronavirus, MERS coronavirus, and porcine deltacoronavirus can take advantage of CTSB/L to enter into host cells (Zhou et al., 2015; Zhang et al., 2019). Likewise, CTSB/L act as SARS-CoV-2 entry-associated proteases, and play a crucial role in promoting SARS-CoV-2 into the host cells (Shang et al., 2020; Trougakos et al., 2021). Therefore, the expressions and distributions of CTSL/B may explain the differences in susceptibility to SARS-CoV-2 infection.

It is well established that cellular and humoral immunity participate in the protection against virus infection. Notably, the pathological process of COVID-19 is probably associated with dysregulation of the immune response, particularly T cells (Qin et al., 2020). Moreover, Merad and Martin (2020) demonstrated that the aberrant and excessive immune cells including monocytes and macrophages played an immune-damaging role in COVID-19. Cancer patients are more susceptible to SARS-CoV-2 infection due to immune dysfunction (Curigliano, 2020; Liang et al., 2020). Furthermore, anticancer therapy including chemotherapeutics or radiotherapy can also induce the systemic immunosuppressive state (Sica and Massarotti, 2017). According to a cohort study of COVID-19 and the Cancer Consortium, patients with cancer and COVID-19 have high mortality (Kuderer et al., 2020). Thus, cancer patients should be paid more attention during the COVID-19 pandemic.

Here, we conducted a profiling analysis of CTSB/L expressions in healthy individuals and patients with pan-cancers by using transcriptomic, genomic, and epigenomic data. Importantly, the correlations between CTSB/L expressions and immune cell infiltration were further analyzed. This study might promote the understanding on susceptible cancer types to SARS-CoV-2 infection and provide a functional foundation for the potential therapy of SARS-CoV-2.

## Materials and Methods

### Expression Profile Analysis

The mRNA and protein expressions of CTSB and CTSL in normal and cancer tissues were analyzed by using the Human Protein Atlas (HPA) database (http://www.proteinatlas.org/) (Uhlen et al., 2015), which consists of six parts: the tissue atlas, single-cell-type atlas, brain atlas, pathology atlas, blood atlas, and cell atlas. The mRNA expression levels of CTSB and CTSL in human cancer cell lines were assessed by the Cancer Cell Line Encyclopedia database (https://portals.broadinstitute.org/ccle) (Barretina et al., 2012).

### Comparisons of Cathepsin B/Cathepsin L Expression Between Cancer and Normal Tissues

The GEPIA2 website (http://gepia2.cancer-pku.cn/) contains RNA sequencing data from The Cancer Genome Atlas (TCGA) and Genotype–Tissue Expression (GTEx) projects (Tang et al., 2019); it was used to compare CTSB and CTSL expressions between cancer and normal tissues. Additionally, the expression levels between the different tumor stages were also analyzed by using GEPIA2.

### Mutation Analysis

Genetic mutation landscapes of CTSB/L in pan-cancers were analyzed by using the c-BioPortal database (https://www.cbioportal.org/) based on TCGA Firehose legacy studies (Cerami et al., 2012; Gao et al., 2013), and the mutation profiles and 3D structures were generated in the mutation module of this database.

### Prognostic Analysis

The association between the CTSB/L expressions and outcomes in different cancers, including the overall survival (OS) and disease-free survival (DFS), was assessed through GEPIA2 using the log-rank test and Cox proportional hazard model.

### Immune Infiltration Analysis

The Tumor Immune Estimation Resource (TIMER) database (http://timer.cistrome.org/) and TISIDB (http://cis.hku.hk/TISIDB/index.php) database were used for the correlation analysis between CTSB/L expressions and immune cell infiltration in diverse cancer types (Li et al., 2017; Ru et al., 2019). Besides, the correlations between the CTSB/L expressions and immune markers in different normal tissues (from TCGA and GTEx) were explored through GEPIA2 using the Spearman method.

## Results

### Expression Profile of Cathepsin B in Normal Human Tissues

**FIGURE 1:**
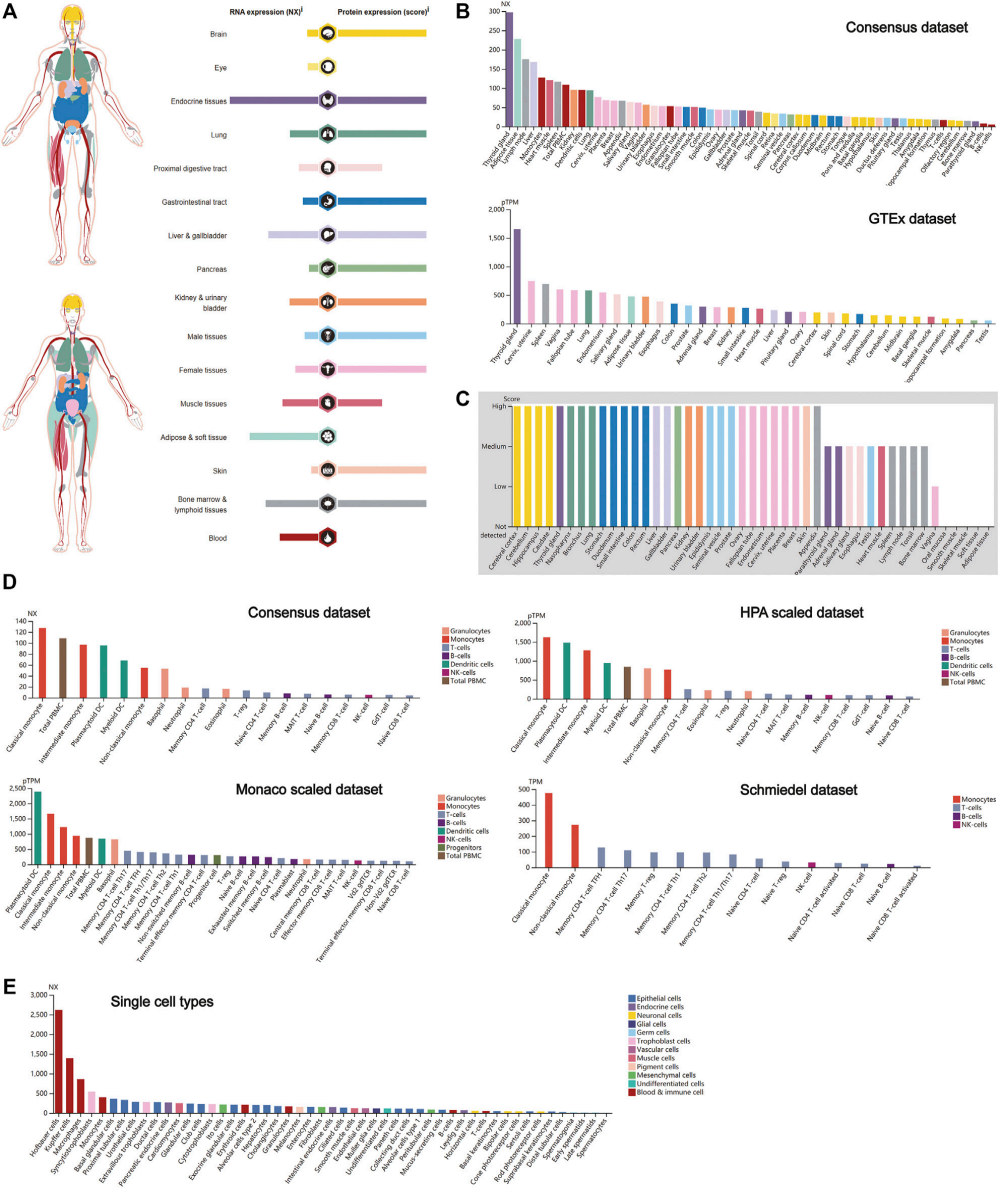
CTSB expression in human tissues. **(A)** Overview of the CTSB expression and distribution in different human tissues. **(B)** The mRNA expression level of CTSB in human tissues which is from the Consensus data set and GTEx data set. **(C)** The protein expression level of CTSB in tissues based on the immunohistochemistry scores. **(D)** The CTSB expression level in human blood cells. **(E)** The CTSB expression level in single cell types.

The expression profiling of CTSB at both the gene transcription and translation levels in human organs and tissues is demonstrated in Figure 1A. It was found that the expression of CTSB mRNA was primarily located in the endocrine, adipose and soft, and bone marrow and lymphoid tissues, whereas the expressional distribution of the CTSB protein was significantly different from its mRNA expression. Except the eye, blood, and adipose and soft tissues, the CTSB protein was generally expressed in all the organs and tissues. Thus, the protein expressions in the endocrine and the bone marrow and lymphoid tissues were consistent with the mRNA expression, while the adipose and soft tissues hardly expressed CTSB protein despite possessing the second highest mRNA expression level. In addition, the expression of CTSB was further verified in the Consensus data set and GTEx data set. In accordance with the results mentioned above, the top three tissues of CTSB mRNA expression in the Consensus data set are the thyroid gland, adipose tissue, and lymph node, respectively. As for the GTEx data set, the thyroid gland had the most CTSB mRNA expression likewise (Figure 1B). In the protein expression data set, it was verified that most organs and tissues had high expressions of CTSB, except for the muscle and adipose tissues (Figure 1C). Furthermore, the mRNA expression of CTSB was investigated in the blood cells in Consensus, Monaco scaled, HPA scaled, and Schmiedel data sets. All the four data sets suggested that CTSB was highly expressed in the monocytes and dendritic cells rather than in the lymphocytes, including the T, B, and NK cells (Figure 1D). The RNA single-cell-type specificity indicated that CTSB was most expressed in the blood and immune cells, especially in the Hofbauer cells, Kupffer cells, macrophages, and monocytes (Figure 1E).

### Expression Profile of Cathepsin L in Normal Human Tissues

**FIGURE 2:**
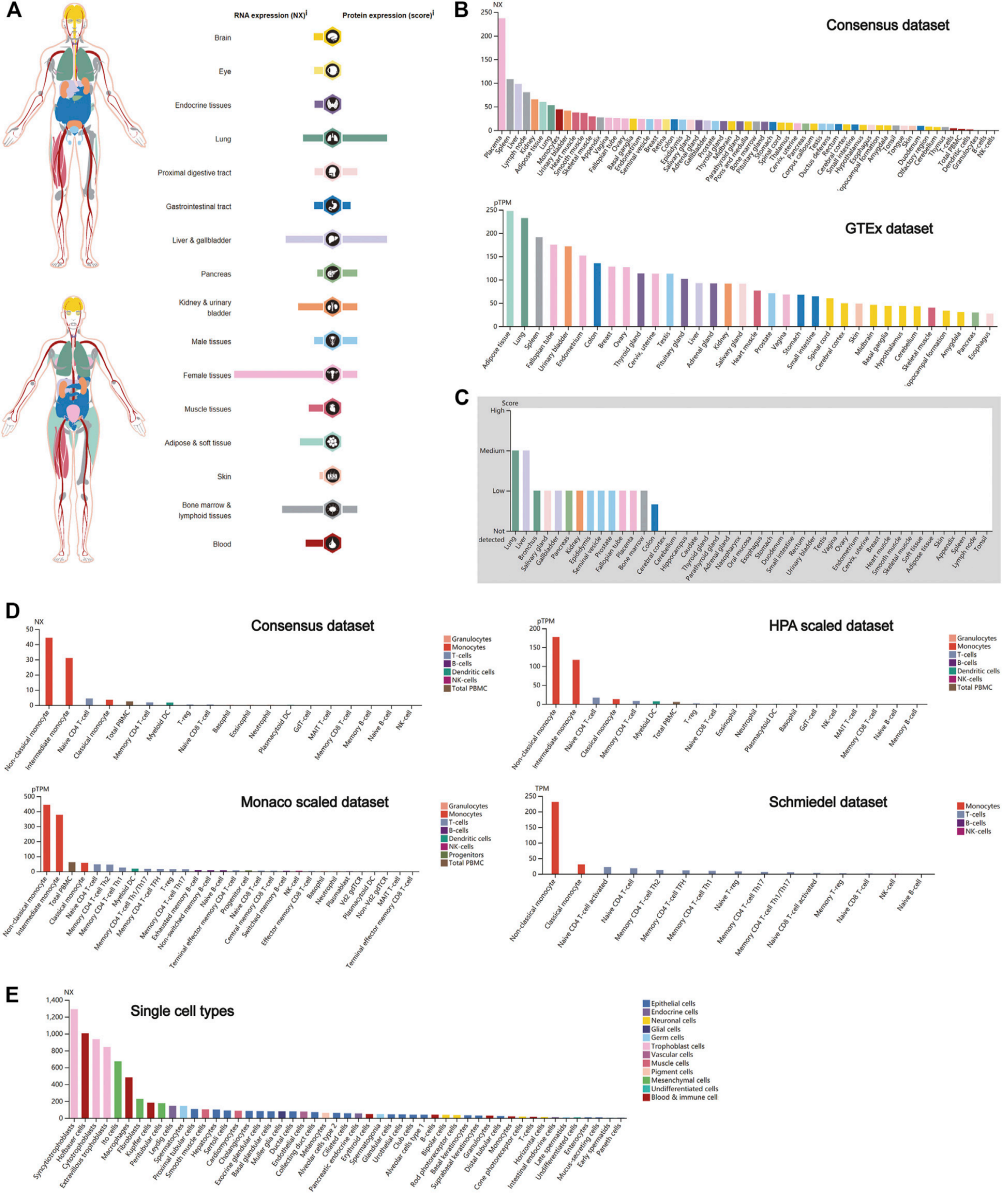
CTSL expression in human tissues. (A**)** CTSL expression and distribution in human tissues. **(B,C)** The mRNA expression level **(B)** and protein expression level **(C)** of CTSL in human tissues. **(D,E)** CTSL expression level in human blood cells **(D)** and single cell types **(E)**.

Similar to CTSB, CTSL also plays a crucial role in promoting SARS-Cov-2 entry into cells. Thus, the expression profile of CTSL was conducted as well. Notably, female tissues have the highest expression level of CTSL mRNA, followed by the bone marrow and lymphoid, and liver and gallbladder tissues (Figure 2A), which roughly resembled the trend in the Consensus and GTEx data sets (Figure 2B). As for the translational level, CTSL was principally enriched in the lung and the liver and gallbladder tissues (Figure 2A), which was further verified in the protein expression data set (Figure 2C). Additionally, the CTSL RNA expression level in monocytes took the leading position in all blood cells (Figure 2D). Finally, the RNA single-cell-type specificity showed that the trophoblast cells had the most mRNA expression of CTSL, including the syncytiotrophoblast, cytotrophoblast, and extravillous trophoblast (Figure 2E).

### Differential Expression of Cathepsin B Between Tumor and Matched Normal Tissues

**FIGURE 3:**
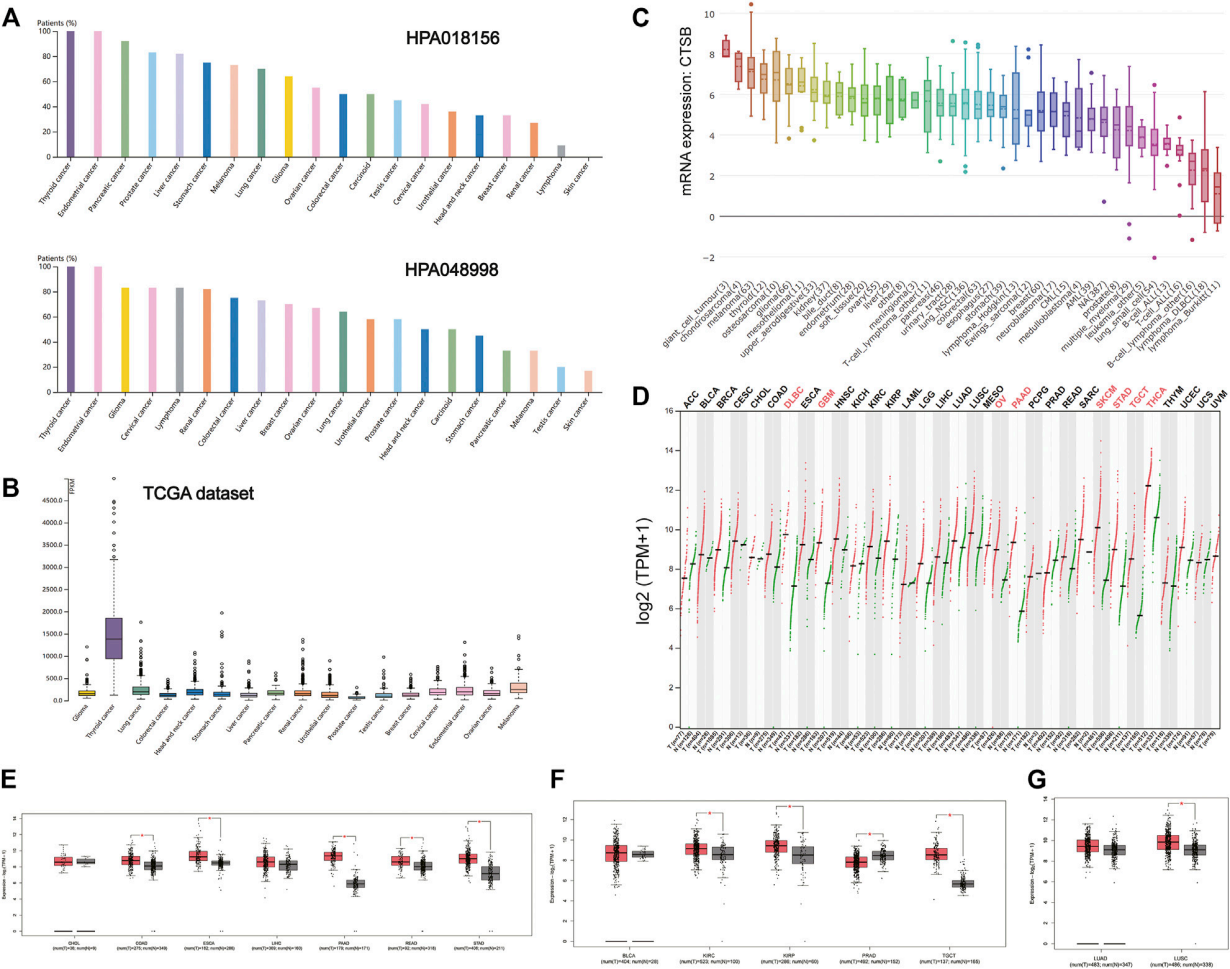
CTSB expression in human cancers. **(A)** The CTSB protein expression level in cancer tissues, which was assessed by HPA018156 and HPA048998 antibodies. **(B)** The CTSB mRNA expression in different cancer tissues. **(C)** The CTSB mRNA expression levels in different human cancer cell lines. **(D)** Bar plot showing CTSB expression levels in multiple cancer tissues and matched normal tissues (TCGA normal + GTEx normal) via GEPIA2. **(E–G)** Boxplot showing differential expression of CTSB between cancers of the digestive, genitourinary, and respiratory systems and matched normal tissues (TCGA normal + GTEx normal) by using GEPIA2. The cutoffs for |log2FC| and *p*-value were set as 0.5 and 0.05, respectively.

There is increasing evidence that patients with cancer are more vulnerable to SARS-Cov-2 infection. It was found that the expressions of CTSB in thyroid and gynecological cancer (especially endometrial cancer) were generally higher than any other cancer types in the two HPA data sets (HPA018156 and HPA048998) and TCGA data set (Figures 3A,B). The mRNA expression of CTSB in cancer cell lines also indicated the thyroid and endometrial cancer had a relatively high CTSB expression (Figure 3C). In addition, the CTSB mRNA expression levels between different cancer tissues and matched normal tissues (TCGA normal + GTEx normal) were compared by using GEPIA2 (Figure 3D). Thereinto, thyroid and ovarian cancers had significantly high CTSB expressions than the matched normal tissues. In the digestive tract, CTSB was overexpressed in colon adenocarcinoma (COAD), esophageal carcinoma (ESCA), pancreatic adenocarcinoma (PAAD), rectum adenocarcinoma (READ), and stomach adenocarcinoma (STAD) rather than cholangiocarcinoma and liver hepatocellular carcinoma (Figure 3E). In the urinary and male reproductive tracts, CTSB was expressed more highly in kidney renal clear-cell carcinoma (KIRC), kidney renal papillary cell carcinoma (KIRP), and testicular germ cell tumors (TGCT) compared with the normal tissues. However, prostate adenocarcinoma (PRAD) had a lower CTSB expression (Figure 3F). As for the respiratory tract, lung squamous cell carcinoma (LUSC) showed a significantly higher expression level of CTSB than the normal lung tissues (Figure 3G).

Furthermore, there were no statistically significant differences in the expression levels among different stages of aerodigestive cancers, except for PAAD, bladder urothelial carcinoma (BLCA), and LUSC for CTSB (Supplementary Figure S1).

### Differential Expression of Cathepsin L Between Tumor and Matched Normal Tissues

**FIGURE 4:**
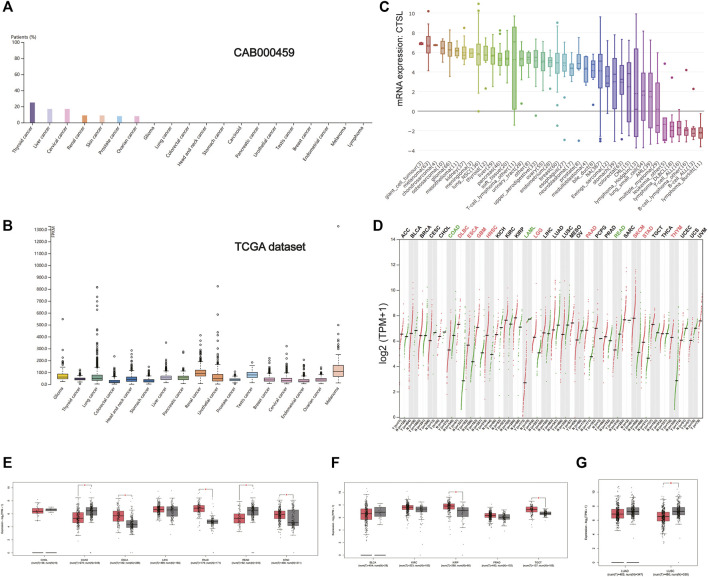
CTSL expression profile in human cancers. **(A)** The CTSL protein expression level in cancer tissues, which was assessed by CAB000459 antibodies. **(B,C)** The CTSL mRNA expression in different cancer tissues **(B)** and different human cancer cell lines **(C)**. **(D)** Bar plot showing CTSL expression levels in multiple cancer tissues and matched normal tissues. **(E–G)** Boxplot showing differential expression of CTSL between cancers of the digestive, genitourinary, and respiratory tracts and matched normal tissues by using GEPIA2. The cutoffs for |log2FC| and *p*-value were set as 0.5 and 0.05, respectively.

As shown in Figure 4A, the expression of CTSL took the leading position in thyroid cancer, followed by liver and cervical cancer in a HPA data set (CAB000459). But in the TCGA data set, the overall CTSL expression remained relatively low in various cancer types with no significant difference (Figure 4B). In the cancer cell lines, the top three cell lines with a high CTSL mRNA expression were giant cell tumor, melanoma, and chondrosarcoma, respectively (Figure 4C).

Likewise, Figure 4D shows the CTSL mRNA expression profiles in different cancer types with their matched normal tissues. In the digestive tract, CTSL was overexpressed in ESCA, PAAD, and STAD while being downregulated in COAD and READ, which was intriguing (Figure 4E). As for the urinary and male reproductive tracts, KIRP and TGCT had a higher level of CTSL expression than the matched normal tissues (Figure 4F). In the respiratory tract, CTSL was significantly downregulated in LUSC (Figure 4G).

Additionally, there existed statistically significant differences in CTSL expression levels among the different stages of ESCA, STAD, BLCA, KIRC, and lung adenocarcinoma (LUAD) (Supplementary Figure S2).

### Mutation Landscape of Cathepsin B/Cathepsin L in Pan-Cancers

**FIGURE 5:**
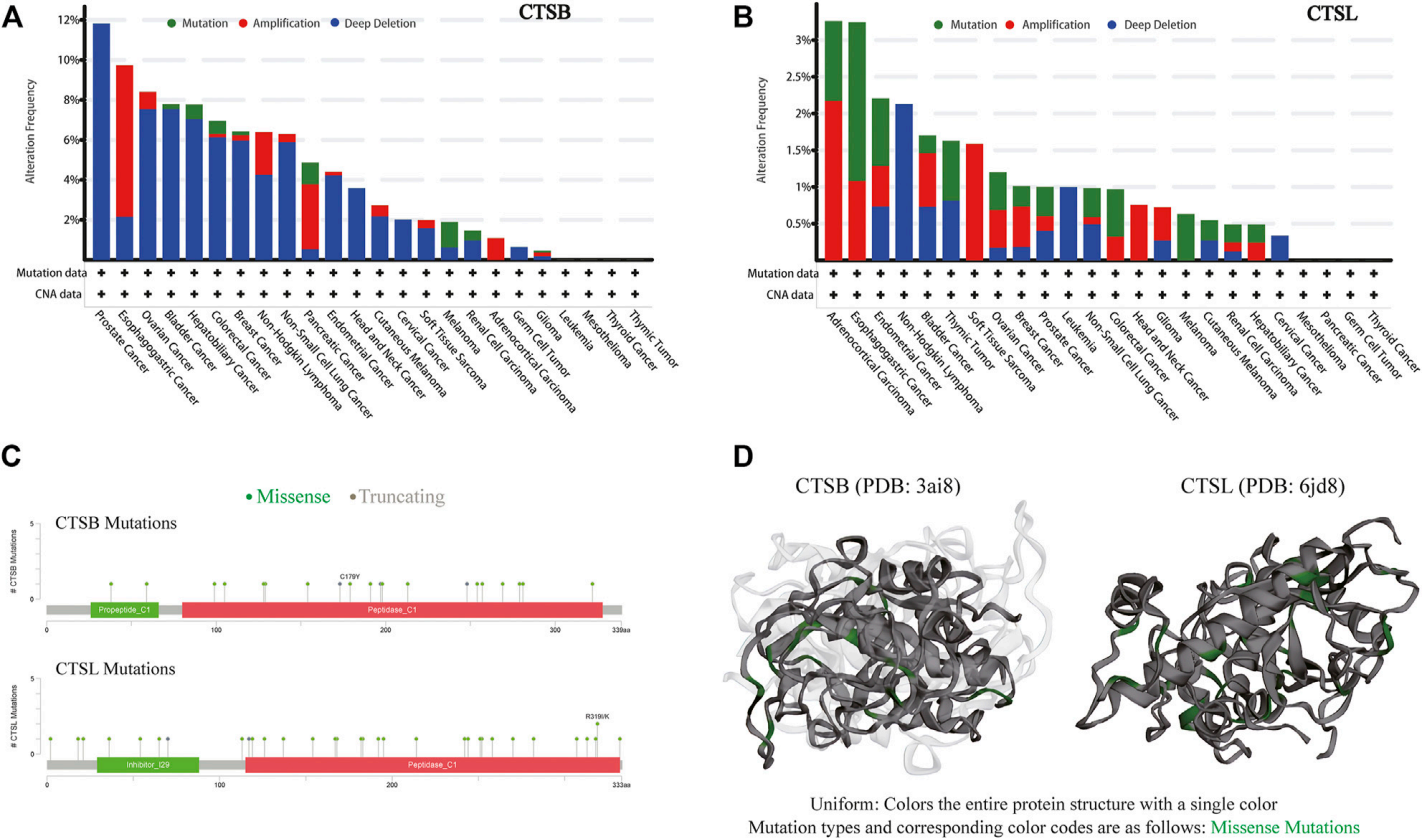
Mutation landscape of CTSB/L in pan-cancers. **(A,B)** The mutation frequency of CTSB **(A)** and CTSL **(B)** based on the TCGA data set from c-BioPortal. **(C)** Natural variants of CTSB/L in cancers. **(D)** The 3D structures of mutation and protein styles of CTSB (PDB 3ai8) and CTSL (PDB 6jd8).

Due to frequent gene mutations in tumors, the mutative status of CTSB and CTSL were analyzed in pan-cancers. The frequency of these two mutated genes was overall less than 10% in the individual cancer types except for around 12% mutated frequency of CTSB in prostate cancer. The top three cancer types with mutated CTSB were prostate, esophagogastric, and ovarian cancers, while those with mutated CTSL were adrenocortical carcinoma, and esophagogastric and endometrial cancers. Additionally, the dominating mutation type of CTSB was deep deletion, while the mutation and amplification were the main mutation types of CTSL (Figures 5A,B). The detailed mutation landscapes of CTSB and CTSL are presented in Figure 5C. Moreover, the three-dimensional crystal structures of the protein and mutation styles are shown in Figure 5D.

### The Prognostic Value of Cathepsin B/Cathepsin L in Pan-Cancers

**FIGURE 6:**
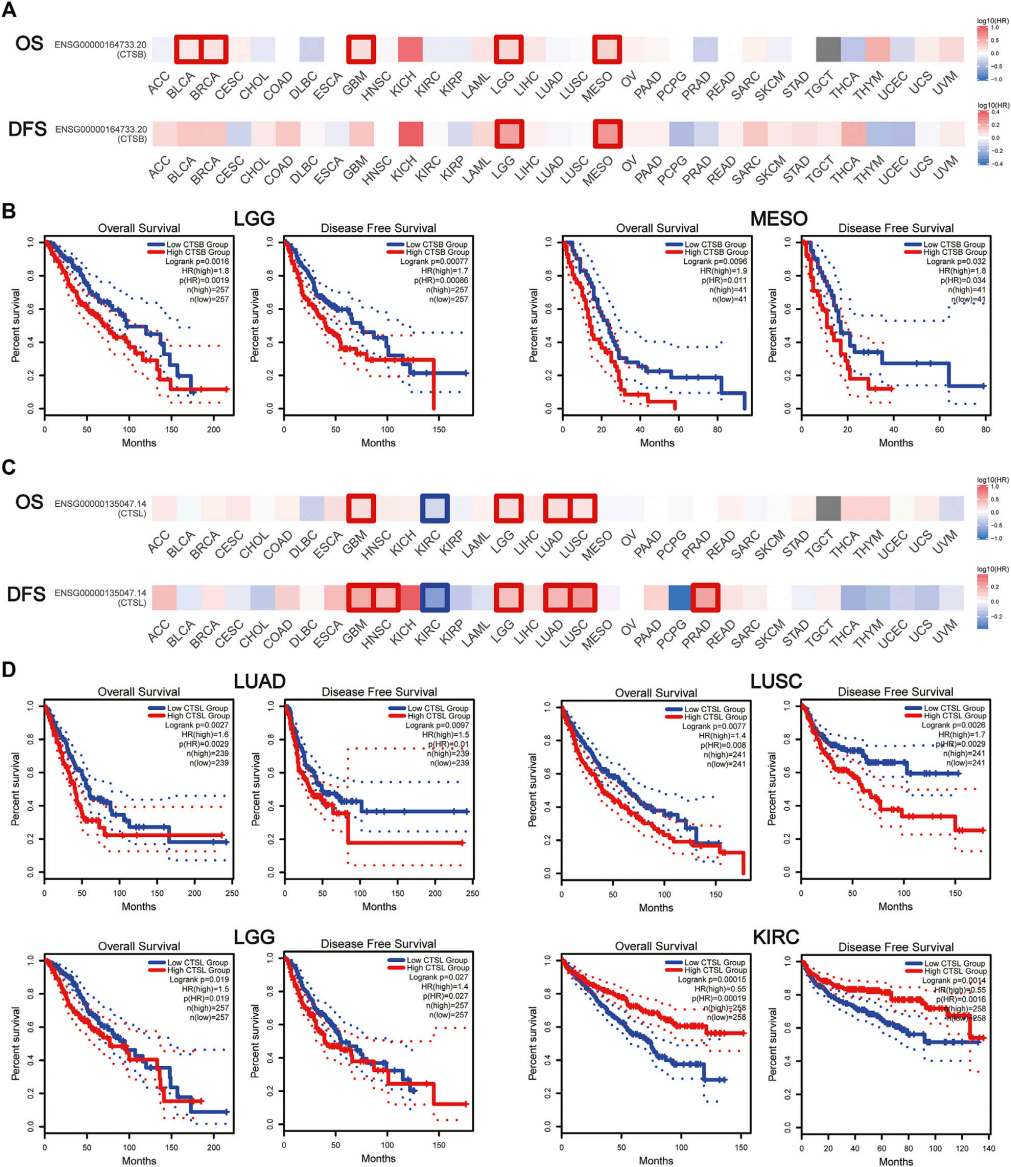
Prognostic value of CTSB/L in pan-cancers. **(A)** Heat map showing the survival significance of CTSB, including OS and DFS, in pan-cancers from GEPIA2 (the red blocks indicate higher risks, blue blocks indicate lower risks; the squares with frames represent *p* < 0.05). **(B)** Kaplan–Meier plots of CTSB for OS and DFS in LGG and MESO. **(C)** Heat map showing the survival significance of CTSL in pan-cancers. **(D)** Kaplan–Meier plots of CTSL in LUAD, LUSC, LGG, and KIRC.

The prognostic value of CTSB and CTSL was further explored in pan-cancers. It was found that CTSB was negatively associated with both OS and DFS in brain low-grade glioma (LGG) and mesothelioma (MESO), and negatively correlated with OS in BLCA, breast invasive carcinoma, and glioblastoma multiforme (GBM) (Figures 6A,B). In addition, CTSL was negatively correlated with both OS and DFS in LUAD, LUSC, LGG, and GBM and negatively correlated with DFS in head and neck squamous-cell carcinoma and PRAD (Figures 6C,D). However, the high level of CTSL in KIRC predicted a prolonged OS and DFS (Figures 6C,D).

### Cathepsin B/Cathepsin L Expression Correlated With Immune Cell Infiltration in Both Cancer and Normal Tissues

**FIGURE 7:**
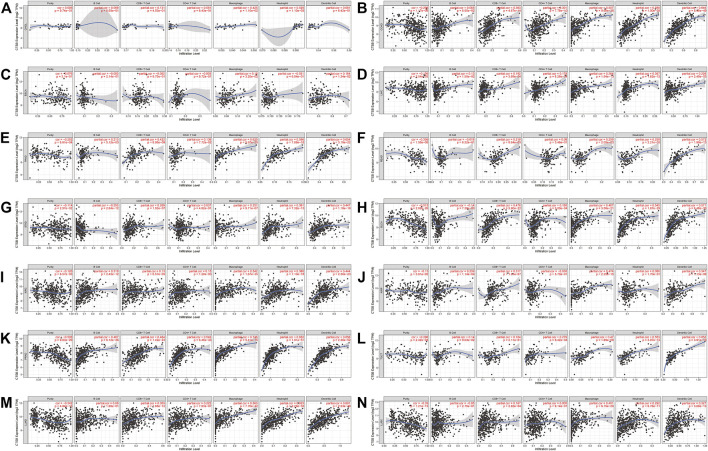
Correlation of CTSB expression and immune infiltration levels in multiple cancers by TIMER database. (**A–G)** The correlations between the CTSB expression and immune cells (B cell, CD4^+^ T cell, CD8^+^ T cell, macrophage, neutrophil, and dendritic cell) in digestive tract cancers. **(H–L)** The correlations between CTSB expression and immune cell infiltration in urinary and male reproductive cancers. **(M–N)** The correlations between CTSB expression and immune cell infiltration in respiratory tract cancers.

Owing to the indispensability of the immune system during antiviral processes, the association between the CTSB expression and immune infiltration level in cancer was investigated by TIMER. Supplementary Figure S3 showed the correlation between CTSB expression and immune infiltration levels in manifold cancer types. Importantly, the expression of CTSB was significantly related to at least three types infiltrating immune cells in thyroid and gynecological cancer. Furthermore, the correlation coefficients in digestive, urogenital, and respiratory cancers are presented in Figures 7A–N. Thereinto, all the cancer types showed a close relation to infiltrating immune cells (at least two types).

Moreover, the association between CTSB and some gene markers of the immune cells in normal tissues was further explored by GEPIA2 (Table 1). It was found that most of the immune cell markers significantly correlated with CTSB expression.

**TABLE1:** The correlations between CTSB and gene markers of immune cells in normal tissues by GEPIA2.

Description	Gene markers	Lung		Esophagus		Stomach		Colon		Liver		Pancreas		Kidney		Bladder		Prostate		Testis	
		Cor	*P*	Cor	*P*	Cor	*P*	Cor	*P*	Cor	*P*	Cor	*P*	Cor	*P*	Cor	*P*	Cor	*P*	Cor	*P*
B cell	CD19	−0.12	**	0.18	**	0.22	**	−0.22	***	0.12	0.14	0.33	***	0.16	*	−0.01	0.96	0.19	*	−0.59	***
	CD79A	0.13	**	0.064	0.31	−0.15	*	−0.19	***	0.48	***	0.33	***	0.42	***	0.053	0.79	0.35	***	0.31	***
CD8^+^ T cell	CD8A	−0.026	0.6	0.41	***	−0.17	*	−0.16	**	0.45	***	0.61	***	0.31	***	0.13	0.51	0.47	***	0.35	***
	CD8B	0.02	0.7	0.38	***	−0.18	**	−0.18	***	0.39	***	0.56	***	0.43	***	0.17	0.38	0.36	***	0.3	***
T cell (general)	CD3D	0.11	*	0.26	***	−0.14	*	−0.18	***	0.47	***	0.43	***	0.3	***	0.1	0.61	0.41	***	0.22	**
	CD3E	0.11	*	0.43	***	−0.11	0.12	−0.19	***	0.42	***	0.63	***	0.4	***	0.075	0.7	0.43	***	0.015	0.85
	CD2	0.12	*	0.36	***	−0.12	0.093	−0.18	***	0.41	***	0.63	***	0.41	***	0.045	0.82	0.45	***	−0.47	***
Macrophage	CD68	0.75	***	0.77	***	0.79	***	0.44	***	0.87	***	0.77	***	0.86	***	0.69	***	0.65	***	0.64	***
	CD11b	0.36	***	0.67	***	0.66	***	0.59	***	0.21	**	0.76	***	0.48	***	0.29	0.14	0.25	**	0.54	***
M1	NOS2	−0.2	***	−0.078	0.21	0.61	***	−0.22	***	0.32	***	0.24	**	0.35	***	-0.082	0.68	0.3	***	0.33	***
	ROS	0.47	***	−0.056	0.37	−0.38	***	−0.26	***	−0.054	0.49	0.25	***	−0.25	**	0.42	*	0.11	0.19	−0.37	***
	IRF5	0.27	***	0.44	***	0.49	***	0.033	0.54	0.15	0.063	0.64	***	−0.31	***	0.096	0.63	0.16	0.046	0.68	***
	COX2	−0.22	***	−0.085	0.17	0.61	***	0.49	***	−0.15	0.067	0.39	***	−0.1	0.21	−0.17	0.39	0.35	***	0.4	***
M2	ARG1	−0.097	0.054	0.05	0.43	0.23	***	0.035	0.52	0.32	***	0.3	***	0.11	0.18	0.62	**	−0.07	0.39	0.1	0.19
	MRC1	0.67	***	0.76	***	0.68	***	0.62	***	0.58	***	0.71	***	0.62	***	0.2	0.32	0.33	***	0.67	***
	CD163	0.61	***	0.74	***	0.52	***	0.56	***	0.74	***	0.74	***	0.37	***	0.29	0.13	0.12	0.16	0.64	***
Monocyte	CD14	0.42	***	0.63	***	0.79	***	0.59	***	0.62	***	0.72	***	0.47	***	0.032	0.87	0.6	***	0.77	***
	CD16A	0.63	***	0.71	***	0.68	***	0.67	***	0.76	***	0.74	***	0.45	***	0.37	0.052	0.52	***	0.7	***
	CD16B	0.027	0.59	0.28	***	0.48	***	0.34	***	0.19	*	0.51	***	0.55	***	0.38	0.048	0.27	***	0.3	***
	CD115	0.62	***	0.51	***	0.41	***	0.47	***	0.76	***	0.74	***	0.56	***	0.36	0.06	0.33	***	0.59	***
Neutrophils	CD15	0.34	***	0.41	***	0.015	0.83	-0.12	*	0.51	***	0.68	***	0.51	***	0.17	0.39	0.35	***	0.46	***
	CD66b	−0.16	**	−0.11	0.085	0.13	0.058	−0.23	***	−0.12	0.13	0.071	0.36	−0.17	*	0.22	0.26	0.041	0.62	−0.29	***
	CD11b	0.36	***	0.67	***	0.66	***	0.59	***	0.21	**	0.76	***	0.48	***	0.29	0.14	0.25	**	0.54	***
Dendritic cell	HLA-DPB1	0.36	***	0.2	**	0.62	***	0.17	**	0.68	***	0.7	***	0.3	***	0.00055	1	0.45	***	0.59	***
	HLA-DQB1	0.085	0.091	−0.08	0.2	0.26	***	−0.017	0.74	0.4	***	0.32	***	0.1	0.19	0.18	0.36	0.23	**	−0.0049	0.95
	HLA-DRA	0.42	***	0.23	***	0.51	***	0.14	**	0.71	***	0.71	***	0.32	***	0.086	0.66	0.54	***	0.57	***
	HLA-DPA1	0.37	***	0.13	*	0.56	***	0.1	0.055	0.69	***	0.64	***	0.32	***	0.054	0.79	0.44	***	0.51	***
	CD1c	0.17	***	0.12	0.058	0.33	***	0.13	*	0.47	***	0.52	***	0.49	***	0.045	0.82	0.43	***	−0.24	**
	CD141	−0.041	0.41	0.081	0.2	0.45	***	0.19	***	0.42	***	0.62	***	0.31	***	0.26	0.18	−0.058	0.48	0.72	***

Cor, R value of Spearman’s correlation. *p < 0.05; **p < 0.01; ***p < 0.001.

**FIGURE 8:**
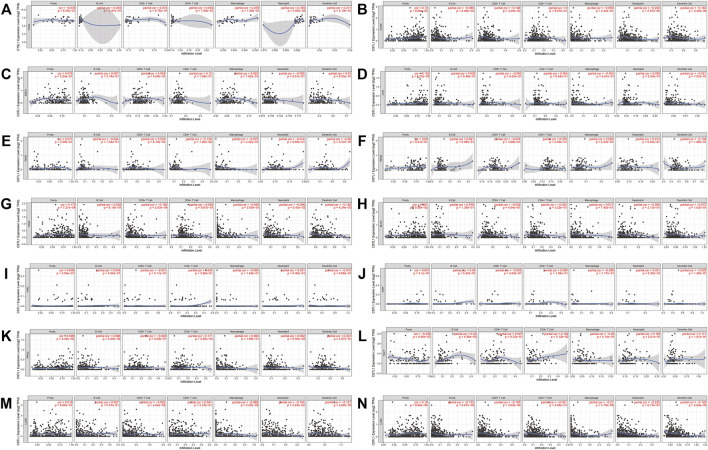
Correlation of CTSL expression and immune infiltration levels in multiple cancers by TIMER database. **(A–G)** The correlations between CTSL expression and immune cell infiltration in digestive tract cancers. **(H–L)** The correlations between CTSL expression and immune cell infiltration in genitourinary cancers. **(M,N)** The correlations between CTSL expression and immune cell infiltration in LUAD and LUSC.

Likewise, the correlation between the CTSL expression and immune infiltration level was also investigated. Supplementary Figure S4 also showed the correlation of the CTSL expression and immune infiltration levels in multiple cancer types. The expressions of CTSL in thyroid and endometrial cancers were related to at least two types of infiltrating immune cells. In digestive, urogenital, and respiratory cancers, only COAD, STAD, LUAD, and LUSC showed a correlation between CTSL and at least two types of infiltrating immune cells (Figures 8A–N).

However, most of the immune cell markers significantly correlated with CTSL expression in normal tissues, which was similar to CTSB (Table 2).

**TABLE2:** The correlations between CTSL and gene markers of immune cells in normal tissues by GEPIA2.

Description	Gene markers	Lung		Esophagus		Stomach		Colon		Liver		Pancreas		Kidney		Bladder		Prostate		Testis	
		Cor	*P*	Cor	*P*	Cor	*P*	Cor	*P*	Cor	*P*	Cor	*P*	Cor	*P*	Cor	*P*	Cor	*P*	Cor	*P*
B cell	CD19	−0.14	**	−0.11	**	0.049	0.48	−0.49	***	0.075	0.35	0.21	**	−0.23	**	−0.018	0.93	0.11	0.16	−0.11	0.17
	CD79A	0.0047	0.93	−0.46	***	−0.44	***	−0.61	***	0.41	***	0.26	***	−0.12	0.14	−0.43	*	0.23	**	0.19	*
CD8^+^ T cell	CD8A	−0.057	0.26	−0.12	**	−0.29	***	−0.55	***	0.4	***	0.45	***	0.014	0.86	−0.51	**	0.17	*	0.041	0.6
	CD8B	−0.055	0.27	−0.11	**	−0.32	***	−0.58	***	0.34	***	0.36	***	−0.032	0.69	−0.61	**8	0.093	0.25	0.16	*
T cell (general)	CD3D	0.022	0.67	−0.33	***	−0.4	***	−0.58	***	0.4	***	0.31	***	−0.03	0.71	−0.57	**	0.18	*	0.33	***
	CD3E	−0.092	0.067	-0.29	***	−0.4	***	-0.6	***	0.31	***	0.39	***	−0.083	0.3	−0.59	**	0.18	*	−0.13	0.091
	CD2	−0.066	0.19	−0.29	***	−0.4	***	−0.59	***	0.31	***	0.43	***	−0.023	0.77	−0.58	**	0.15	0.058	−0.18	*
Macrophage	CD68	0.46	***	−0.048	0.21	0.34	***	−0.073	0.17	0.87	***	0.65	***	0.1	0.2	−0.22	0.26	0.67	***	0.43	***
	CD11b	0.32	***	0.78	***	0.74	***	0.67	***	0.36	***	0.53	***	−0.17	*	0.65	***	0.094	0.25	0.2	*
M1	NOS2	0.0038	0.94	0.44	***	0.5	***	-0.62	***	0.14	0.07	0.15	*	0.25	**	−0.16	0.43	0.044	0.59	0.32	***
	ROS	0.16	**	0.044	0.26	−0.34	***	-0.66	***	−0.093	0.24	0.32	***	0.45	***	−0.048	0.81	0.28	***	−0.057	0.47
	COX2	−0.016	0.75	0.5	***	0.75	***	0.8	***	−0.086	0.28	0.22	**	0.22	**	0.027	0.89	0.26	**	−0.013	0.87
M2	ARG1	0.12	*	−0.28	***	0.33	***	0.14	**	0.26	***	0.29	***	−0.29	***	−0.11	0.58	−0.12	0.14	0.12	0.12
	MRC1	0.32	***	0.74	***	0.69	***	0.35	***	0.62	***	0.7	***	0.086	0.28	0.78	***	0.51	***	0.32	***
	CD163	052	***	0.73	***	0.68	***	0.44	***	0.77	***	0.72	***	−0.072	0.37	0.64	***	0.19	*	0.36	***
Monocyte	CD14	0.47	***	0.79	***	0.62	***	0.21	***	0.58	***	0.63	***	0.21	**	0.56	**	0.56	***	0.32	***
	CD16A	0.37	***	0.69	***	0.53	***	0.35	***	0.74	***	0.59	***	0.045	0.58	0.43	*	0.45	***	0.29	***
	CD16B	0.08	0.11	0.26	***	0.36	***	0.2	***	0.19	*	0.33	***	0.065	0.42	−0.025	0.9	0.21	**	−0.067	0.39
	CD115	0.32	***	0.56	***	0.23	***	0.18	***	0.72	***	0.53	***	0.028	0.72	0.33	0.088	0.29	***	0.21	**
Neutrophils	CD15	0.21	***	0.63	***	−0.18	**	−0.59	***	0.58	***	0.53	***	0.12	0.13	0.2	0.3	0.55	***	0.44	***
	CD66b	−0.004	0.94	0.02	0.61	0.12	0.09	−0.56	***	−0.057	0.47	−0.038	0.62	−0.058	0.47	−0.2	0.31	−0.068	0.41	−0.012	0.88
	CD11b	0.32	***	0.78	***	0.74	***	0.67	***	0.36	***	0.53	***	−0.17	*	0.65	***	0.094	0.25	0.2	*
Dendritic cell	HLA-DPB1	0.11	*	0.43	***	0.3	***	−0.094	0.078	0.57	***	0.47	***	0.2	*	−0.48	**	0.38	***	0.24	**
	HLA-DQB1	0.0037	0.94	−0.066	0.089	0.021	0.76	−0.17	**	0.34	***	0.19	*	−0.085	0.29	−0.46	*	0.11	0.16	−0.0055	0.94
	HLA-DRA	0.19	***	0.26	***	0.14	*	−0.18	***	0.61	***	0.46	***	0.29	***	−0.45	*	0.49	***	0.15	0.05
	HLA-DPA1	0.13	*	0.38	***	0.25	**	−0.16	**	0.6	***	0.42	***	0.25	**	−0.49	**	0.44	***	0.17	*
	CD1c	−0.094	0.06	−0.35	***	−0.026	0.71	−0.02	0.71	0.33	***	0.33	***	0.022	0.78	−0.48	**	0.27	***	−0.078	0.32
	CD141	0.012	0.8	−0.28	***	0.54	***	0.33		0.6	***	0.51	***	0.15	0.055	−0.15	0.44	0.36	***	0.35	***

Cor, R value of Spearman’s correlation. *p < 0.05; **p < 0.01; ***p < 0.001.

**FIGURE 9:**
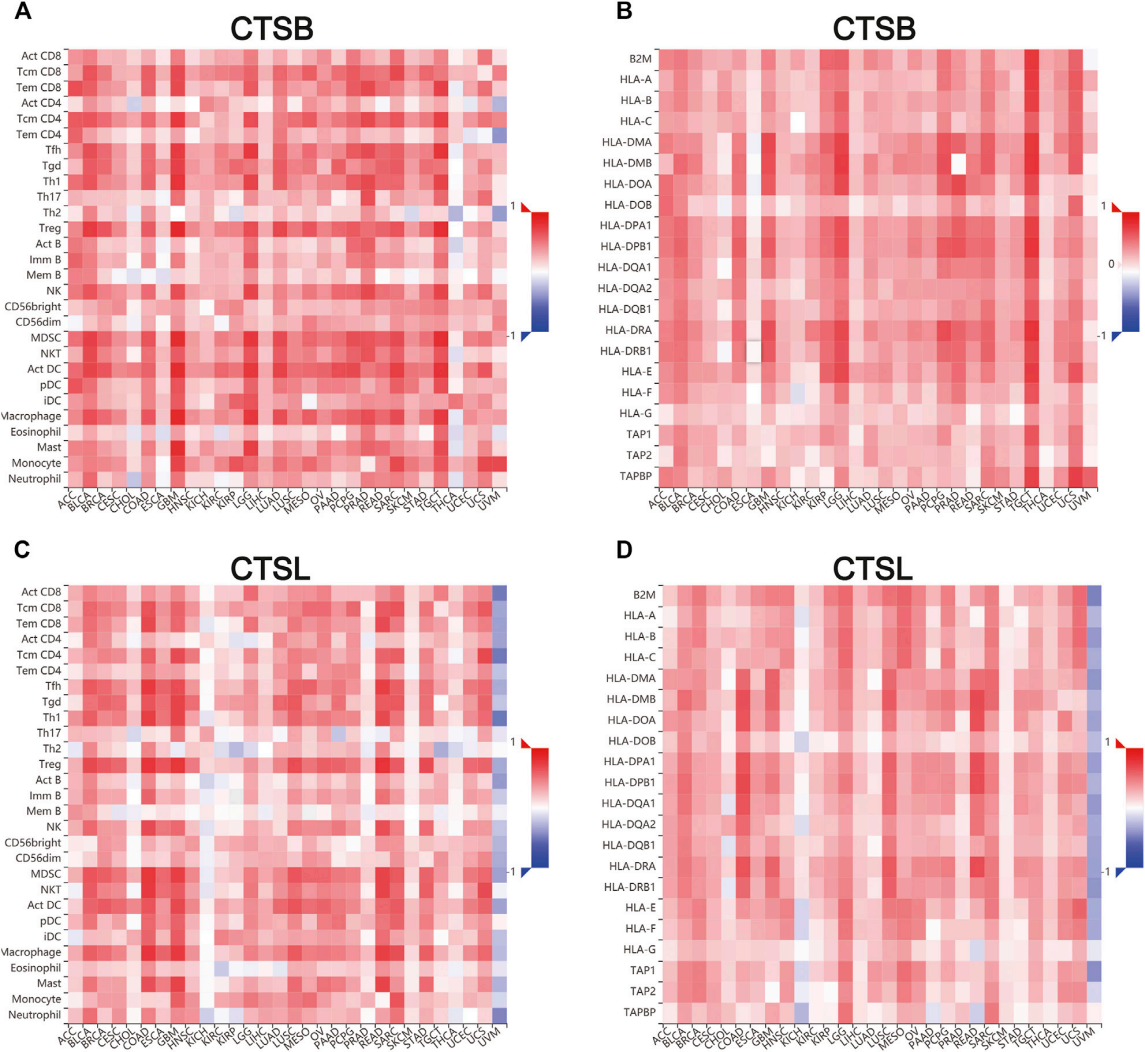
Correlation of CTSB/L expression with tumor–immune system in pan-cancers determined by the TISIDB database. **(A,B)** The overview of correlation between CTSB expression and immune cells, MHC molecules in pan-cancers. **(C,D)** The correlations between CTSL expression and immune cells, and MHC molecules in pan-cancers.

Additionally, we further verified the correlation between CTSB/L and tumor-infiltrating immune cells, and major histocompatibility complex (MHC) molecules in another database TISDB, which has more detailed classifications of the immune cells (Figures 9A–D). The results showed that CTSB/L had been positively related to most infiltrating immune cells and MHC molecules in almost all cancer types. However, there was a negative association between CTSL and the infiltrating immune cells in uveal melanoma.

## Discussion

The worldwide pandemic of COVID-19 has been a rigorous challenge for public health and social stability. Therefore, it is urgent to identify susceptible individuals and prevent them from infection. Furthermore, cancer patients should get more attention owing to high mortality caused by some severe symptoms, such as respiratory failure and multi-organ failure (Liang et al., 2020).

Evidence has shown that CTSB/L play a critical role in the process of coronavirus infecting host cells. Bollavaram et al. (2021) found that there existed multiple sites on SARS-CoV-2 spike protein that are prone to proteolysis by CTSB/L. Thus, inhibition of CTSB/L can prevent SARS-CoV-2 infection. There are three CTSL inhibitors showing potent efficiency in blocking SARS-CoV-2 infection (Smieszek et al., 2020; Ma et al., 2022). However, these preclinical studies should be further investigated in patients. According to the results mentioned above, the expressions of CTSB/L were widely expressed in human tissues. It is speculated that the leakage of pulmonary vessels during acute respiratory distress syndrome could lead to the spread of SARS-CoV-2 in the circulation and eventually infect organs with high CTSB/L expressions (Gkogkou et al., 2020; Ma et al., 2022).

Moreover, CTSB/L have been reported to serve as oncogenes (Li et al., 2020), which is in accordance with our result that expressions of CTSB/L in cancer are generally higher than the matched normal tissues. Additionally, we found that CTSB had a potential prognostic value in LGG and MESO, while CTSL might predict a poor prognosis in LUAD, LUSC, LGG, and GBM (Figure 6). However, the prognostic value of CTSB/L was analyzed through the TCGA database at the transcriptome level, which should be further verified in a larger sample size at the translational level. Furthermore, the underlying mechanisms also need to be explored. Importantly, CTSB/L had significantly higher expressions in thyroid and gynecological cancer than any other cancer type (Figures 3, 4). Kim et al. (2020) found that CTSB was a promising prognostic marker of thyroid cancer due to its function of enhancing the migration capacity of cancer cells. It was also identified that CTSB played a cancer-promoting role in endometrial and cervical cancers (Wu et al., 2012; Bao et al., 2013). As for CTSL, Zhang et al. (2014) reported that the upregulated CTSL was a biomarker of invasion and metastasis in ovarian cancer. Overall, it is suggested that the overexpression of CTSB/L not only predicts a poor prognosis of cancers but also increases the susceptibility of SARS-CoV-2, leading to unsatisfied clinical outcomes of cancer patients. Nevertheless, whether SARS-CoV-2 can infect tumor tissues merits further investigation considering the limited biopsy samples.

Although the expressions of CTSB/L in aerodigestive and genitourinary tract cancers are not as high as found in thyroid and gynecological cancers, there remains a high risk of SARS-CoV-2 infection in these cancers. The increased potential of being infected is probably attributed to these organs being exposed to the external environment, which provides favorable routes for SARS-CoV-2 transmission (Patel et al., 2020; He et al., 2021). Therefore, it is also critical to pay attention to patients with aerodigestive and genitourinary tract cancers to prevent SARS-CoV-2 infection.

On the one hand, the infiltration of numerous immune cells often occurs at SARS-CoV-2 infection sites (Xu et al., 2020; Yuki et al., 2020). On the other hand, cancer patients usually harbor dysfunctional immune cells in their circulation or the tumor microenvironment, such as T cells, NK cells, and dendritic cells (Saxena and Bhardwaj, 2018; Scheper et al., 2019; Zhang and Liu, 2020). Moreover, tumor-associated macrophages that are classified into the M1 and M2 phenotypes constitute an indispensable part of the dynamic tumor microenvironment (Cassetta and Pollard, 2020). Thereinto, M2 populations play a carcinogenic function of repressing anti-cancer immune response, while M1 ones exert the opposite function (Pan et.al., 2020). Nonetheless, the host immune response to SARS-CoV-2 in cancer patients has not been fully demonstrated. Our results show that the immune cell infiltration was correlated with CTSB expression in most cancer types and CTSL expression in a part of the cancer types (COAD, STAD, LUAD, and LUSC) (Figures 7, 8, respectively). When infection occurs, the immune cells move into the infection and position against SARS-CoV-2. This hypothesis may partially explain the decreased number of lymphocytes and monocytes in the circulation in the patients with COVID-19 (Qin et al., 2020). Although CTSB/L indicates an increased level of immune cell infiltration in various cancers, the prognosis might not be better because the immune cells tend to be blunt and dysfunctional (Huang et al., 2020). We provided an overview of the association between CTSB/L and immune cell infiltration, but the specific association needs further experimental evidence due to the predominant immune cells varying from cancer to cancer (Kohchiyama et al., 1987; Kurebayashi et al., 2018; Huang and Fu, 2019; Karamitopoulou, 2019).

However, there still are some limitations about our research. For example, this study mainly relies on bioinformatics analysis, and a validation cohort to validate our findings is lacking. Also there is a lack of experimental data and mechanism research in understanding the link between the cancer data on SARS-CoV-2 and the potential role for CTSB/L; further experimental data is therefore needed.

Taken together, our study reveals the expression and distribution of two novel SARS-CoV-2 entries CTSB/L in different tissues/organs, and the differential expression, mutation landscape and specifically prognostic significance across different cancer types, and immune implications, although these need to be further proven in future studies. Our current work certainly might provide some useful implication for understanding the pandemic of SARS-CoV-2.

## Data Availability

The original contributions presented in the study are included in the article/Supplementary Material, and further inquiries can be directed to the corresponding authors.

## Abbreviations

SARS-CoV-2, severe acute respiratory syndrome coronavirus 2; COVID-19, coronavirus diseases 2019; CTSB, cathepsin B; CTSL, cathepsin L; HPA, Human Protein Atlas; CCLE, Cancer Cell Line Encyclopedia; GTEx, Genotype–Tissue Expression; TCGA, The Cancer Genome Atlas; COAD, colon adenocarcinoma; ESCA, esophageal carcinoma; PAAD, pancreatic adenocarcinoma; READ, rectum adenocarcinoma; STAD, stomach adenocarcinoma; CHOL, cholangiocarcinoma; LIHC, liver hepatocellular carcinoma; KIRC, kidney renal clear cell carcinoma; KIRP, kidney renal papillary cell carcinoma; TGCT, testicular germ cell tumors; PRAD, prostate adenocarcinoma; LUSC, lung squamous-cell carcinoma; BLCA, bladder urothelial carcinoma; LGG, low-grade glioma; MESO, mesothelioma; BRCA, breast invasive carcinoma; GBM, glioblastoma multiforme; LUAD, lung adenocarcinoma; HNSC, head and neck squamous-cell carcinoma; UVM, uveal melanoma.
