# Supplementary figures and images for: Comprehensive and Integrative Analysis of Two Novel SARS-CoV-2 Entry Associated Proteases CTSB and CTSL in Healthy Individuals and Cancer Patients
Source: Front Bioeng Biotechnol. 2022 Jan 26;10:780751. doi: 10.3389/fbioe.2022.780751 (PMC8826559; doi:10.3389/fbioe.2022.780751)

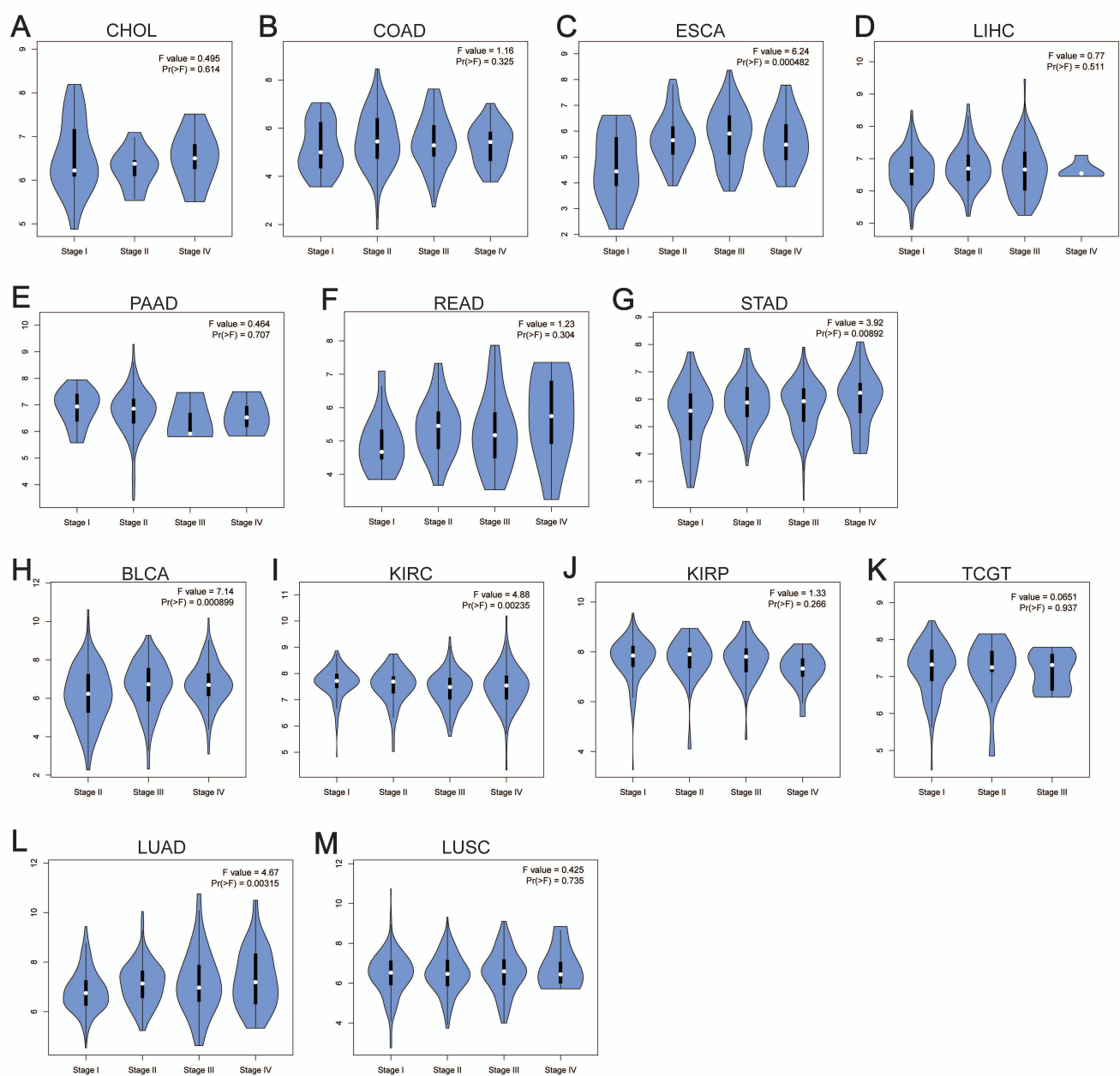

Supplement: Supplementary file 1 [file DataSheet2.PDF]

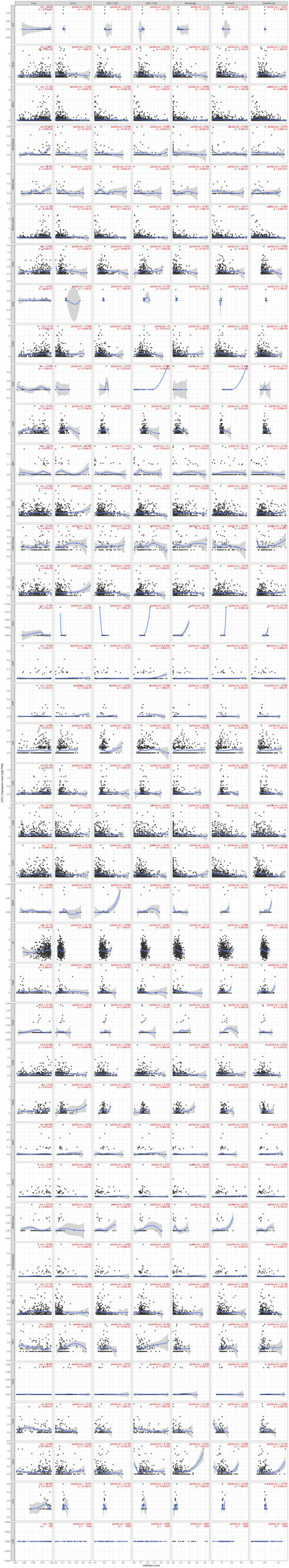

Supplement: Supplementary file 2 [file DataSheet4.PDF]

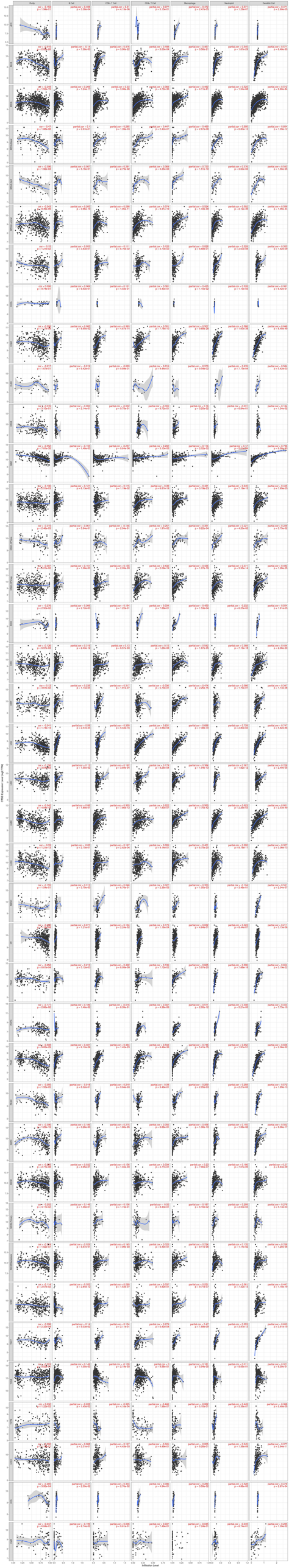

Supplement: Supplementary file 3 [file DataSheet3.PDF]

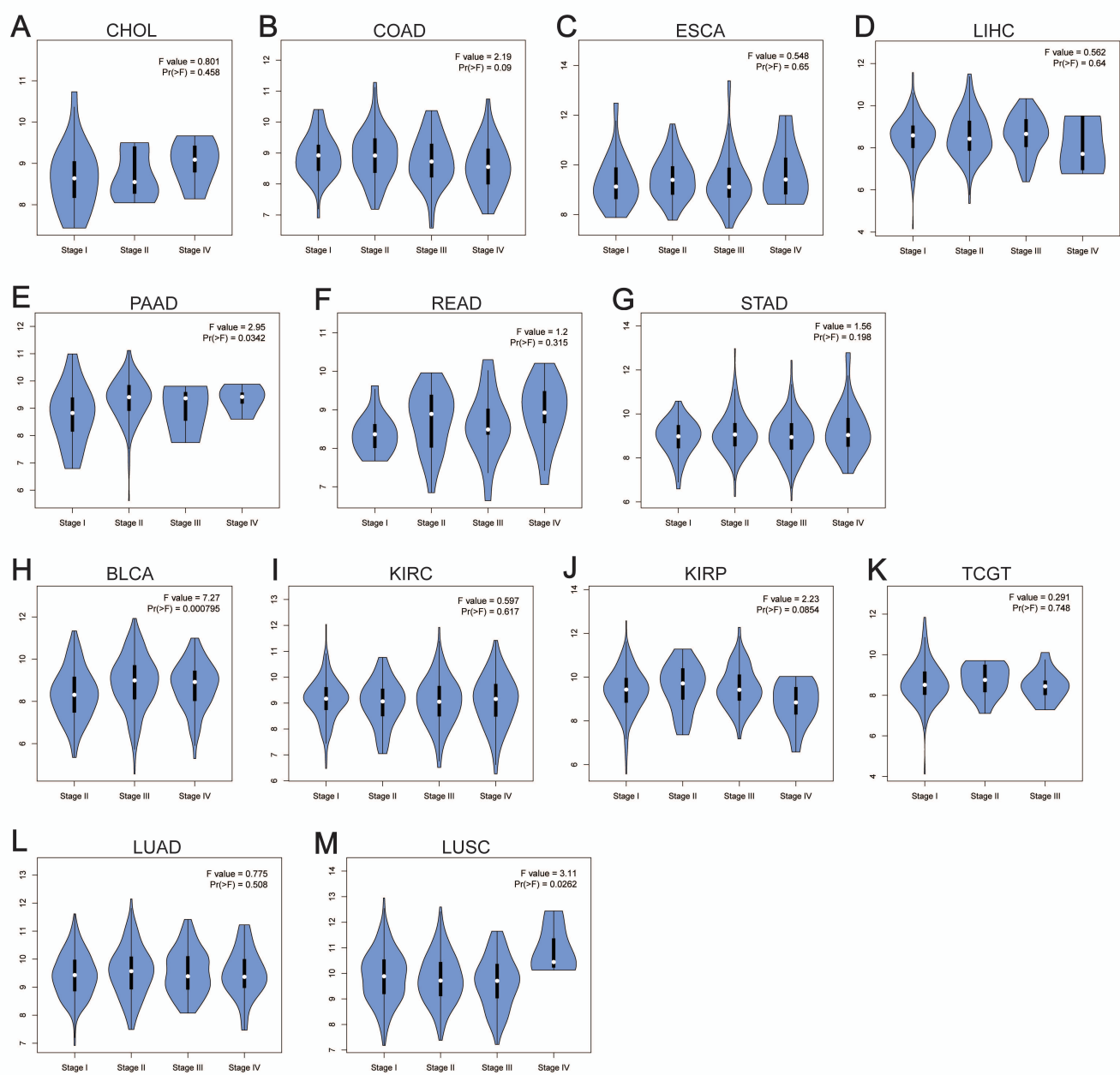

Supplement: Supplementary file 4 [file DataSheet1.PDF]
